# Patterns of medical pluralism among adults: results from the 2001 National Health Interview Survey in Taiwan

**DOI:** 10.1186/1472-6963-10-191

**Published:** 2010-07-06

**Authors:** Chun-Chuan Shih, Yi-Chang Su, Chien-Chang Liao, Jaung-Geng Lin

**Affiliations:** 1 School of Chinese Medicine, China Medical University, Taichung, Taiwan; 2 Institute of Environmental Health, College of Public Health, China Medical University, Taichung, Taiwan; 3 Management Office for Health Data, China Medical University Hospital, Taichung, Taiwan

## Abstract

**Background:**

Medical pluralism (MP) can be defined as the employment of more than one medical system or the use of both conventional and complementary and alternative medicine (CAM) for health and illness. A population-based survey and linkage with medical records was conducted to investigate MP amongst the Taiwanese population. Previous research suggests an increasing use of CAM worldwide.

**Methods:**

We collected demographic data, socioeconomic information, and details about lifestyle and health behaviours from the 2001 Taiwan National Health Interview Survey. The medical records of interviewees were obtained from National Health Insurance claims data with informed consent. In this study, MP was defined as using both Western medicine and traditional Chinese medicine (TCM) services in 2001. The odds ratio (OR) and 95% confidence interval (CI) were estimated for factors associated with adopting MP in univariate and multiple logistic regression.

**Results:**

Among 12,604 eligible participants, 32.5% adopted MP. Being female (OR = 1.44, 95% CI = 1.30 - 1.61) and young (OR = 1.38, 95% CI = 1.15 - 1.66) were factors associated with adopting MP in the multiple logistic regression. People with healthy lifestyles (OR = 1.35, 95% CI = 1.19 - 1.53) were more likely to adopt MP than those with unhealthy lifestyles. Compared with people who had not used folk therapy within the past month, people who used folk therapy were more likely to adopt MP. The OR of adopting MP was higher in people who lived in highly urbanised areas as compared with those living in areas with a low degree of urbanisation. Living in an area with a high density of TCM physicians (OR = 2.19, 95% CI = 1.69 - 2.84) was the strongest predictor for adopting MP.

**Conclusion:**

MP is common in Taiwan. Sociodemographic factors, unhealthy lifestyle, use of folk therapy, and living in areas with a high density of TCM physicians are all associated with MP. People who had factors associated with the adoption of MP may be at risk for adverse health effects from interactions between TCM herbal medicine and WM pharmaceuticals.

## Background

The increasing use of complementary and alternative medicine (CAM) in children and adults has been investigated in Eastern and Western countries [1-16], and this interesting phenomenon has attracted physicians' attention [17-19]. Many physicians recognize that their patients are interested in using CAM therapies, some physicians are very interested in learning more about CAM [17]. Eisenberg suggested that medical doctors should ask about their patients' use of unconventional therapy whenever they obtain a medical history [1]. The utilisation of traditional Chinese medicine (TCM) is not uncommon in Taiwan [11,16,20-25] and other Asian countries [4,11,15,16,20,22,26]. It was estimated that the one-month, one-year, and six-year utilization of TCM service in Taiwan were 10.4%, 28.4%, and 62.5%, respectively [23,25]. Western medicine (WM) is believed to be useful all over the world, while the traditional medicine of India, China, Korea, Taiwan and other countries are also well established and commonly used [4,9-11,13,16,20,22,25-28].

Medical pluralism (MP) can be defined as the employment of more than one medical system or the use of both conventional medicine and CAM for health and illness [29]. Previous researches have documented the increased adopting MP for China, Sri Lanka, and the United States [28-33]. A small-scale study conducted with self-reported medical questionnaires reported MP in Taiwan more than ten years ago [21]. However, the recent patterns of MP and associated factors in Taiwan and other countries need to be investigated more closely.

Demographic characteristics and factors related to an individual's health status are associated with CAM use [34]. For example, Shih et al. [25] reported that being female, highly educated, or having a self-reported poor health status were predictive factors associated with TCM use. To the best of our knowledge, there have been no studies reporting factors associated with MP. Using the National Health Interview Survey (NHIS) and data from the National Health Insurance (NHI) system, this study reports on factors associated with adopting MP among adults aged 18 years and older in Taiwan.

## Methods

### Study design and data collection

In 2001, the National Health Research Institute and the Bureau of Health Promotion of Taiwan conducted a nationwide NHIS using face-to-face questionnaire interviews. The population of Taiwan is approximately 23 million, distributed throughout 7 cities and 18 counties. The 2001 NHIS included a representative sample of 22,121 interviews from the non-institutional population. The interviewees were all residents, and each interview was performed at the subject's home. All subjects interviewed were selected from the household census. With the standardised face-to-face questionnaire interview, the NHIS used a multistage, stratified sampling scheme to collect a representative sample of the population of Taiwan. The 2001 NHIS was a cross-sectional survey, and the sampling and measurement details are described elsewhere [16,35]. The response rate was 94% for individual subjects. At the end of the NHIS, the participants were asked for permission to access their NHI records for research purposes [36]. Among all the participants, 88% gave consent, and 12% denied consent. No discrepancy in age or gender was found between individuals who consented and those who refused [36].

Both the 2001 NHIS and the 2001 NHI claims data were used in this study. With the informed consent of eligible participants, the 2001 NHIS data were linked to the 2001 NHI claims data. The NHI database included ambulatory care, inpatient care, dental service, prescription drugs, registration file, and scrambled identification numbers released for public access. All Taiwanese residents are eligible to receive health service under the NHI system. The NHI is a type of compulsory insurance for ensuring all residents can obtain equal and complete health care. As of 2008, the NHI covered more than 99% of all Taiwan residents. The data contained information on gender, date of birth, encrypted patient identification numbers, dates of admission and discharge, International Classification of Diseases (ICD) codes of discharge diagnoses (up to 5), operative procedures (up to 5), and discharge costs (i.e., expenditures) by admission.

All eligible participants in this study were 18 years or older at the time, and their use of WM and/or TCM was drawn from the NHI claims data. The 2001 NHIS included questions relating to sociodemographic factors, self-perceived health status, self-reported height and weight, medical services utilisation, lifestyles and heath behaviours.

### Definition and measures

We have defined MP, TCM, and folk therapy (FT) in the following section.

#### Medical pluralism (MP)

In this study, we defined those adopting MP as people who had used both WM and TCM at least one time each in 2001. The use of WM or TCM was reported in NHI medical records. The visits for TCM and WM ambulatory care were considered as the use of TCM and WM, respectively. In Taiwan, TCM is included in the NHI system only in the case of ambulatory care, not for inpatient care. In addition, only licensed TCM physicians qualify for reimbursement from the NHI. Importantly, internal medicine, surgical medicine, and emergency medicine records were included in the WM category. Because TCM-hospitalisation is not covered under the NHI, we did not report inpatient TCM service in this study. At the end of 2001, there were 2 public TCM hospitals, 42 private TCM hospitals and 2,544 private TCM clinics providing TCM ambulatory care [11].

#### Traditional Chinese medicine (TCM)

TCM includes herbal medicine, acupuncture, moxibustion, bone reduction, traditional trauma treatment, traditional dislocation treatment, traditional fracture treatment, Tuina, Baguan, and other therapies. TCM practitioners are registered TCM physicians and practice in a hospital or clinic. TCM in Taiwan is legal, and TCM physicians can advertise the medical benefits of TCM according to medical law in Taiwan.

#### Folk therapy (FT)

In this study, FT use is defined as utilisation of folk therapy within the past month. The types of FT included Gua Sha (scraping skin), Tuina (massage and kneading), Baguan (vacuum bottle therapy), bone setting, spine alignment, Qigong, divination, written charms, shaman consultation, talismans, incense ash, and other related therapies. There may be some overlap between what is considered TCM versus FT. The difference, however, lies in the legality. FT practitioners are not registered physicians and do not practice in a hospital or clinic. Legislation from the Department of Health in Taiwan has declared that FT practitioners cannot claim any medical benefits for FT. They are not registered physicians and do not have any certifications or legal licenses. FT practitioners do not work in clinical settings in Taiwan.

The definition and classification of urbanisation and unhealthy lifestyles are as follows.

#### Urbanisation

There are 359 townships and city districts in Taiwan. We calculated the population density (persons/km^2^) by dividing the population (persons) by the area (km^2^) for each of these administrative units. The first, second, and third tertiles were considered as areas of low, moderate and high urbanisation, respectively [37]. We calculated the density of physicians (physicians/10,000 persons) by using the number of physicians per 10,000 persons for each of the administrative units. The first, second, and third tertiles were considered as areas of low, moderate, and high physician density, respectively.

#### Unhealthy lifestyle

A high prevalence of cigarette smoking, alcohol drinking, and betel quid chewing was found in Taiwan [38]. In this study, people with a habit of tobacco smoking and/or alcohol drinking and/or areca chewing were considered to have unhealthy lifestyles that are associated with cancer and other diseases [38-40].

In accordance with regulations from the Department of Health in Taiwan, stroke, chronic obstructive pulmonary disease, cancer, chronic renal failure, autoimmune disease, chronic psychological disease, congenital metabolic disorders, rare diseases, and organ transplantation were considered severe diseases in this study.

### Statistical analysis

The eligible study subjects were divided into two groups: one group of MP users and another of non-MP users. The distribution of categorical variables such as sociodemographic factors, lifestyles, disease states, and health behaviours in each group were compared using a Chi-square test. The odds ratio (OR) and 95% confidence interval (CI) were estimated for factors associated with adopting MP using multiple logistic regression. We used Hosmer-Lemeshow statistics [41] for assessing the goodness of fit of the logistic regression model. We also calculated the rate of adoption of MP in relation to a variety of participant characteristics.

## Results

We excluded 5,993 participants below 18 years of age. We also excluded 2,047 adult participants whose claims were denied, according to their NHI medical records. Further, we excluded those that did not use WM or TCM in 2001 (n = 1,477). However, there were no significant differences in the age (p = 0.06) or the sex (p = 0.96) of participants versus non-participants. Finally, there were 12,604 eligible participants included in this study, 32.5% of whom adopted MP (Table 1). Among the 12,416 people that used WM in 2001, 33.0% of them also used TCM. On the other hand, 4,094 of the 4,282 TCM-visiting patients used WM (95.6%) in 2001 (not shown in the tables).

**Table 1:** Sociodemographic factors of study participants by medical pluralism

	Medical pluralism					
			
	No		Yes		Total	
	N = 8510		N = 4094		N = 12604	
Sex	n	(%)	n	(%)	n	(%)
Male	4397	(72.9)	1637	(27.1)	6034	(100)
Female	4113	(62.6)	2457	(37.4)	6570	(100)
Age, years						
18-29	2242	(66.9)	1110	(33.1)	3352	(100)
30-49	3503	(65.7)	1830	(34.3)	5333	(100)
50-64	1559	(68.0)	733	(32.0)	2292	(100)
≥ 65	1206	(74.1)	421	(25.9)	1627	(100)
Education, years						
0	795	(71.8)	313	(28.2)	1108	(100)
1-9	2867	(67.2)	1399	(32.8)	4266	(100)
10-12	2537	(67.0)	1249	(33.0)	3786	(100)
≥ 13	2305	(67.1)	1131	(32.9)	3436	(100)
Occupation						
Unemployed	2219	(70.8)	916	(29.2)	3135	(100)
Homemaker	1335	(64.8)	726	(35.2)	2061	(100)
Skilled, Unskilled	3797	(66.7)	1893	(33.3)	5690	(100)
Profession	1128	(67.5)	1893	(32.5)	1672	(100)
Family income, NTDs						
< 30,000	1639	(69.1)	732	(30.9)	2371	(100)
30,000-49,999	1857	(66.9)	920	(33.1)	2777	(100)
50000-99999	3383	(66.1)	1732	(33.9)	5115	(100)
≥ 100,000	1567	(69.8)	677	(30.2)	2244	(100)
Urbanization						
Low	768	(74.1)	269	(25.9)	1037	(100)
Moderate	1030	(69.8)	445	(30.2)	1475	(100)
High	6712	(66.5)	3380	(33.5)	10092	(100)

NTDs, new Taiwan dollars

The Chi-square test showed that women in Taiwan had a higher rate than men of adopting MP (37.4% vs. 27.1%, p < 0.0001). People aged 30 - 49 years had a higher rate of MP adoption than those aged 65 years and above. The corresponding rate was higher in people with higher education than in people without higher education (32.9% vs. 28.2%, p < 0.0001). A significant difference in adopting MP could also be found based on occupation (p < 0.0001), family income (p < 0.0001), and degree of urbanisation (p < 0.0001) in the Chi-square tests.

Table 2 showed that the rate of adopting MP was higher in people with no unhealthy lifestyle factors than in those who had at least two unhealthy lifestyle factors (35.8% vs. 25.2%, p < 0.0001). The Chi-square test also showed that people who used FT were more likely to adopt MP than people with no FT use (49.0% vs. 31.4%, p < 0.0001). Living in an area with a high density of TCM physicians (p < 0.0001) was associated with adopting MP. The Spearman's correlation coefficient showed that highly urbanised areas were also areas with a high density of TCM physicians (r = 0.62, p < 0.0001) (not shown in the tables).

**Table 2:** Health behaviours and physician density factors of study participants by medical pluralism

	Medical pluralism					
			
	No		Yes		Total	
	N = 8510		N = 4094		N = 12604	
Have unhealthy lifestyle						
No	4766	(64.2)	2662	(35.8)	7428	(100)
One	1698	(69.6)	740	(30.4)	2438	(100)
Two or three	2043	(74.8)	690	(25.2)	2733	(100)
Severe disease						
No	8269	(67.4)	4001	(32.6)	12270	(100)
Yes	241	(72.2)	93	(27.8)	334	(100)
Have regular health checkup						
No	7046	(67.7)	3356	(32.3)	10402	(100)
Yes	1464	(66.5)	738	(33.5)	2202	(100)
Use of folk therapy						
No	8123	(68.6)	3723	(31.4)	11846	(100)
Yes	383	(51.0)	368	(49.0)	751	(100)
Density of TCM physicians						
Low	568	(82.1)	124	(17.9)	692	(100)
Moderate	1995	(69.5)	874	(30.5)	2869	(100)
High	5947	(65.8)	3096	(34.2)	9043	(100)
Density of WM physicians						
Low	845	(70.8)	348	(29.2)	1193	(100)
Moderate	1869	(68.4)	865	(31.6)	2734	(100)
High	5796	(66.8)	2881	(33.2)	8677	(100)

TCM, traditional Chinese medicine; WM, Western medicine

Table 3 showed the OR and 95% CI of factors associated with MP in the univariate and multiple logistic regressions. The Hosmer-Lemeshow statistics provided a p-value of 0.67 (Chi-square = 5.83), indicating that the overall model fit is good. Compared with men, women were more likely to adopt MP (OR = 1.44, 95% CI = 1.30 - 1.61). Age, education, occupation and family income were also associated with adopting MP in the multiple logistic regression. People who lived in an area with a high density of TCM physicians had a higher likelihood of adopting MP than those who lived in areas with a low density of TCM physicians (OR = 2.19, 95% CI = 1.69 - 2.84). The OR was higher in people that had used FT than in people with no use of FT (OR = 1.90, 95% CI = 1.63 - 2.23). Having no unhealthy lifestyle factors was also associated with adopting MP (OR = 1.35, 95% CI = 1.19 - 1.53). However, the density of WM physicians was not associated with adopting MP in the multiple logistic regression.

**Table 3:** Odds ratios and 95% confidence intervals of factors associated with medical pluralism in the univariate and multiple logistic regression

	Multivariate	
	
	OR	(95% CI)
Sex		
Male	1.00	(reference)
Female	1.44	(1.30-1.61)
Age, years		
18-29	1.38	(1.15-1.66)
30-49	1.44	(1.20-1.72)
50-64	1.33	(1.11-1.58)
≥ 65	1.00	(reference)
Education, years		
0	1.00	(reference)
1-9	1.25	(1.02-1.53)
10-12	1.13	(0.91-1.40)
≥ 13	1.08	(0.86-1.36)
Occupation		
Unemployed	1.00	(reference)
Homemaker	0.84	(0.72-0.98)
Skilled, Unskilled	0.93	(0.80-1.08)
Profession	0.95	(0.79-1.14)
Family income, NTDs		
< 30,000	1.19	(1.03-1.37)
30,000-49,999	1.23	(1.08-1.40)
50000-99999	1.20	(1.07-1.34)
≥ 100,000	1.00	(reference)
Urbanization		
Low	1.00	(reference)
Moderate	1.15	(0.94-1.41)
High	0.96	(0.80-1.16)
Density of TCM physicians		
Low	1.00	(reference)
Moderate	1.80	(1.40-2.31)
High	2.19	(1.69-2.84)
Density of WM physicians		
Low	1.00	(reference)
Moderate	1.03	(0.87-1.22)
High	0.90	(0.76-1.06)
Use of folk therapy		
No	1.00	(reference)
Yes	1.90	(1.63-2.23)
Have unhealthy lifestyle		
No	1.35	(1.19-1.53)
One	1.22	(1.07-1.39)
Two or three	1.00	(reference)

NTDs, new Taiwan dollars; TCM, traditional Chinese medicine; WM, Western medicine

The characteristics of people with a high rate of MP adoption are shown in Table 4. Women aged 30 - 49 years who had no unhealthy lifestyle factors, used FT, had a family income of 30,000 - 49,999 NTD, and lived in an area with a high density of TCM physicians had the highest rate of MP adoption (64.7%).

**Table 4:** Characteristics of people adopting medical pluralism

Number	Female	30-49 years	No unhealthy lifestyle	Use of folk therapy	Family income = 30000-49,999	Live in high density of TCM physicians	MP use, %	(95% CI)
2457	Yes						37.4	(36.2-38.6)
1086	Yes	Yes					38.5	(36.7-40.3)
911	Yes	Yes	Yes				39.0	(37.0-40.9)
95	Yes	Yes	Yes	Yes			54.9	(47.5-62.3)
27	Yes	Yes	Yes	Yes	Yes		64.3	(60.0-68.6)
22	Yes	Yes	Yes	Yes	Yes	Yes	64.7	(48.6-80.8)

NTDs, new Taiwan dollars; TCM, traditional Chinese medicine

## Discussion

From the results of this study, we found that the MP adoption rate was as high as 32.5% among adults who had medical visits in 2001 in Taiwan. Sociodemographic factors were significant factors associated with adopting MP. The most significant factor for adopting MP was living in an area with a high density of TCM physicians. These data challenge the biomedical profession's previous assumption that a single biomedical system defines our society's health care practices [35]. To the best of our knowledge, our study is the first large-scale study to report on the prevalence of adopting MP and the associated factors in Taiwan.

Demographic factors such as age and sex were found to be associated with a patient's choice of WM or TCM [11,21]. Young and middle-aged adults were more likely than older people to use TCM in Taiwan; this trend also extends to Caucasians [9,16,24]. A previous study suggested that young people seek more effective ways to improve their well-being and health and to relieve disease symptoms [23]. As a result, it is reasonable that younger people had a higher likelihood of using MP than older people in this study. One study indicated that females' intake of TCM herbal medicine is helpful in maintaining regular menstruation [42]. This is one explanation that may help explain why females were more likely to adopt MP.

Socioeconomic factors were important factors associated with the use of medical services [43-51]. People with a higher socioeconomic status (SES) were more likely to use CAM or TCM than those with a low SES. Many studies also reported that a higher SES was associated with using biomedical health services and physical examinations [16,21,22,46-50]. Those with a higher SES had a greater likelihood of using medical services without insurance payment than people with a low SES [16,21-23]. We failed to find, however, an association between SES and MP adoption based on the results of this study. This is mainly because TCM was covered in the NHI, and people in Taiwan do not generally need to pay much additional money to use medical services.

In this study, we found that people living in highly urbanised areas had a higher OR of adopting MP. Generally speaking, urbanisation is positively correlated with the density of medical services. People living in highly urbanised areas had more opportunities to access various conventional or non-conventional therapies. Moreover, people living in areas with a low degree of urbanisation also tended to have poorer access to medical services. Previous studies have found that high levels of urbanisation are associated with a higher volume of medical use in Taiwan [23,25].

Economic expansion is a major determinant of physician supply and medical utilisation [52,53]. The relationship between the increasing use of TCM and the growth in the number of TCM physicians in Taiwan has been investigated [54]. In Taiwan, Chinese medical care services exhibit a physician-induced demand situation and the increase in Chinese medical care expenditures was caused by the growth of Chinese medical physicians from 1996 to 1999 [54]. We found that living in areas with a high density of TCM physicians was the strongest predictor of adopting MP. However, living in areas with a high density of WM physicians was not associated with adopting MP in this study.

Highly urbanised areas were also areas with a high density of TCM physicians in this study (data shown in the results section). The lack of TCM physicians in rural areas is also an important medical problem for Taiwan. Specifically, 105 of 359 (29.2%) towns in Taiwan do not have any TCM physicians. It is reasonable to posit that an increase in the supply of WM or TCM services may increase medical use. Furthermore, in this study, one out of three WM-using people used TCM in 2001, while 95% of TCM-using people used WM (data shown in the results section). This revealed that people who lived in areas with an increased density of TCM physicians had a greater opportunity to utilise TCM services[25].

FT users were more likely to adopt MP than FT non-users in our study. WM is the mainstream in Taiwan and other developed countries, though TCM is legal in Taiwan and covered by NHI. Some parts of FT are similar to TCM. However, practitioners of FT are not licensed and cannot declare that their therapy has any medical benefits because of the medical legislation in Taiwan [16].

Our study results implied that people who adopted MP were potentially at risk of the combined use of Chinese herbal medicine and biomedical pharmaceuticals. Specifically, the combined use of warfarin and danshen may result in over-anticoagulation and bleeding complications [55,56]. Further, Page et al. [57] found that there was a twofold elevation in prothrombin time and international normalised ratio after taking dong quai and warfarin concurrently for 4 weeks. It has been reported that the concurrent use of ephedra and theophylline may lead to hypertension [58]. Intake of ginseng may increase the side effects of steroids [59,60]. The increasing use of CAM has also been investigated in the United States and other Western countries [1-10,12,13,15]. This study points to the need to continue monitoring and investigating the interaction between CAM and WM in Western countries, a suggestion that has been mentioned in previous studies [29,32].

In this study, we found that a high percentage (88%) of adults gave consent for their NHI records and questionnaire results to be linked. People who refused to give consent for accessing their NHI medical records may have done so because of confidentiality and privacy concerns. One plausible explanation is that some of the participants may have been hesitant to give consent because of ineffective communication or a lack of trust between themselves and the interviewers [36].

The fact that we used data from a large survey that represented the non-institutionalised Taiwan population is the main strength of this study. The study still has some limitations, however. One limitation of this study is that it is a cross-sectional perspective on MP and does not provide any information about whether it is increasing or decreasing over time or whether the characteristics of adopters are changing over time. Recall bias has been given considerable attention in textbooks and methodological research because of its potential to jeopardise the validity of epidemiologic results [61]. We also understand that the results of this study may be biased if respondents misreported their sociodemographic and health behaviours. Another potential limitation is the definition of MP that was adopted and its consistency with the definition used in previous research [29,32].

## Conclusion

We found that adopting MP is common in Taiwan, and we found several predictive sociodemographic factors. People living in highly urbanised areas had more opportunities to access various conventional and non-conventional therapies. The types of diseases that prompt people to adopt MP need to be determined in further studies. This study investigated the high degree of MP use, which points to the need to investigate the interaction between WM pharmaceuticals and TCM herbal medicine and the effects of their combined use on health in further studies. People may need to be screened for factors associated with the adoption of MP because they may be at risk for adverse health effects from TCM herbal interactions with WM pharmaceuticals.

## Abbreviations

CAM: complementary and alternative medicine; FT: folk therapy; NHI: National Health Insurance; NHIS: National Health Interview Survey; MP: medical pluralism; OR: odds ratio; CI: confidence interval; SES: socioeconomic status; TCM: traditional Chinese Medicine; WM: Western medicine.

## Competing interests

The authors declare that they have no competing interests.

## Authors' contributions

CCS and CCL developed the study concept, conducted data analyses and initiated the draft. YCS and JGL helped the acquisition of data and revised the manuscript critically for the important content. CCL served as a methodological consultant and a statistical consultant. All authors read and approved the final manuscript.

## Pre-publication history

The pre-publication history for this paper can be accessed here:

http://www.biomedcentral.com/1472-6963/10/191/prepub
